# A geometrical approach to control and controllability of nonlinear dynamical networks

**DOI:** 10.1038/ncomms11323

**Published:** 2016-04-14

**Authors:** Le-Zhi Wang, Ri-Qi Su, Zi-Gang Huang, Xiao Wang, Wen-Xu Wang, Celso Grebogi, Ying-Cheng Lai

**Affiliations:** 1 School of Electrical, Computer and Energy Engineering, Arizona State University, 650 E. Tyler Mall, Tempe, Arizona 85287-5706, USA; 2 Institute of Computational Physics and Complex Systems, Lanzhou University, 222 S. Tianshui Road, Lanzhou, Gansu 730000, China; 3 School of Biological and Health Systems Engineering, Arizona State University, 621 E. Tyler Mall, Tempe, Arizona 85287-9709, USA; 4 School of Systems Science, Beijing Normal University, 19 Xinjiekou Outer Street, Beijing, 100875, China; 5 Institute for Complex Systems and Mathematical Biology, King's College, Meston Walk, University of Aberdeen, Aberdeen AB24 3UE, UK; 6 Institute for Complex Systems and Mathematical Biology, King's College, University of Aberdeen, Meston Walk, Aberdeen AB24 3UE, UK; 7 Department of Physics, Arizona State University, 550 E Tyler Drive, Tempe, Arizona 85287-1504, USA

## Abstract

In spite of the recent interest and advances in linear controllability of complex networks, controlling nonlinear network dynamics remains an outstanding problem. Here we develop an experimentally feasible control framework for nonlinear dynamical networks that exhibit multistability. The control objective is to apply parameter perturbation to drive the system from one attractor to another, assuming that the former is undesired and the latter is desired. To make our framework practically meaningful, we consider restricted parameter perturbation by imposing two constraints: it must be experimentally realizable and applied only temporarily. We introduce the concept of attractor network, which allows us to formulate a quantifiable controllability framework for nonlinear dynamical networks: a network is more controllable if the attractor network is more strongly connected. We test our control framework using examples from various models of experimental gene regulatory networks and demonstrate the beneficial role of noise in facilitating control.

## Abstract (web summary)

Complex networks, including physical, biological and social systems are ubiquitous, but understanding of how to control them is elusive. Here Wang *et al*. develop a framework based on the concept of attractor networks to facilitate the study of controllability of nonlinear dynamics in complex systems.

## Graphical abstract

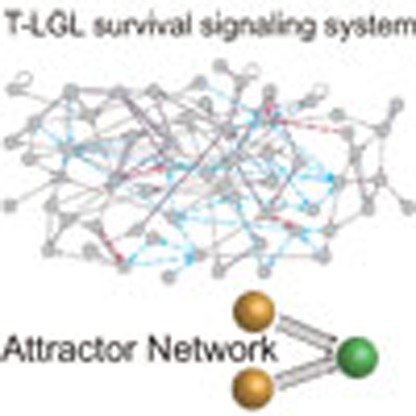

Nonlinear dynamical processes are ubiquitous in natural and engineering systems. Significant difficulties arise when such processes are coupled with the complex network topology, especially in terms of control. In spite of progress in understanding, analysing and predicting the behaviours of large complex networks, to formulate an effective framework to control nonlinear dynamical networks has remained to be an outstanding problem in interdisciplinary research.

In recent years, the traditional control and graph theories have been exploited to determine the linear controllability of complex networks^1^,^2^,^3^,^4^,^5^,^6^,^7^,^8^,^9^. The efforts have led to a quantitative understanding of the interplay between controllability and network structure. In particular, in the structural controllability framework^1^, the concept of maximum matching was proposed to determine and quantify whether a directed complex network can be driven from an arbitrary initial state to any desired state in finite time. It was found that the degree distribution of the network is critical to its controllability^1^. Subsequently, an alternative framework, the exact controllability framework^2^, was developed to extend the linear controllability analysis to networks with arbitrary structures and link weight distributions. In this framework, one uses the principle of maximum geometrical multiplicity of the network spectrum to determine the minimum set of driver nodes required to fully control the network. The mathematical underpinning of the structural and exact controllability frameworks is the classic Kalman's rank condition^10^, whose applicability is limited to linear dynamical networks. Nonlinear control theory based on the Lie brackets^11^ and a recent work to extend the linear controllability and observability theory to nonlinear networks with symmetry^9^ notwithstanding, to establish a general mathematical controllability framework for complex and nonlinear dynamical networks appear not realistic at the present.

Owing to the high dimensionality of nonlinear dynamical networks and the rich variety of behaviours that they entail, it may be prohibitively difficult to develop a control framework that is universally applicable to different kinds of network dynamics. In particular, the classic definition of linear controllability, that is, a network system is controllable if it can be driven from an arbitrary initial state to an arbitrary final state in finite time, is generally not applicable to nonlinear dynamical networks. Instead, controlling collective dynamical behaviours may be feasible^12^,^13^. Our idea is that, for nonlinear dynamical networks, control strategies may need to be specific and system dependent. The purpose of this paper is to articulate control strategies and develop a controllability framework for nonlinear networks that exhibit multistability. A defining characteristic of such systems is that there are multiple coexisting attractors in the phase space^14^,^15^,^16^. The goal is to drive the system from one attractor to another using physically meaningful, temporary and finite parameter perturbation, assuming that the system is likely to evolve into an undesired state (attractor) or the system is already in such a state and one wishes to implement control to bring the system out of the undesired state and steer it into a desired one.

In biology, nonlinear dynamical networks with multiple attractors have been employed to understand fundamental phenomena such as cancer emergence^17^, cell fate differentiation^18^,^19^,^20^,^21^ and cell cycle control^22^,^23^. For example, Boolean network models were used to study gene evolution^24^, attractor number variation with asynchronous stochastic updating^25^ and gene expression in the state space^19^. Another approach is to abstract key regulation genetic networks^26^,^27^ (or motifs) from all associated interactions, and to employ synthetic biology to modify, control and finally understand the biological mechanisms within these complicated systems^18^,^22^. An earlier application of this approach led to a good understanding of the ubiquitous phenomenon of bistability in biological systems^28^, where there are typically limit cycle attractors and, during cell cycle control, noise can trigger a differentiation process by driving the system from a limit circle to another steady-state attractor^18^. Generally, there are two candidate mechanisms for transition or switching between different attractors^20^: through signals transmitted within cells and noise, which were demonstrated recently using synthetic genetic circuits^29^. More recently, a detailed numerical study revealed how signal-induced bifurcations in a tri-stable genetic circuit can lead to transitions among different cell types^21^.

In this paper, we develop a control and controllability framework for nonlinear dynamical networks based on the concept of attractor networks^30^. An attractor network is defined in the phase space of the underlying nonlinear system, in which each node represents an attractor and a directed edge from one node to another indicates that the system can be driven from the former to the latter using experimentally feasible, temporary and finite parameter changes. A well-connected attractor network implies a strong feasibility that the system can be controlled to reach a desired attractor. The connectivity of the attractor network can then be used to characterize the controllability of the original nonlinear dynamical network. More specifically, for a given pair of attractors, the relative weight of the shortest path is the number of accessible control parameters whose adjustments can lead to the attractor transition as specified by the path. We use gene regulatory networks (GRNs) to demonstrate the practicality of our control framework, which includes low-dimensional, experimentally realizable synthetic gene circuits and a realistic T-cell cancer network of 60 nodes. A finding is that noise can facilitate control by reducing the required amplitude of the control signal. We emphasize that the development of our nonlinear control framework is based entirely on physical considerations, rendering feasible experimental validation.

## Results

A complex, nonlinear dynamical network of *N* variables can be described by a set of *N*-coupled differential equations:


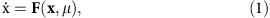


where **x**∈**R**^*N*^ denotes the *N*-dimensional state variable, **F**(**x**, *μ*) is the nonlinear vector field and *μ*∈**R**^*M*^ represents the set of coupling parameters. In a GRN, the nodal dynamics is typically one-dimensional. For simplicity, we assume that this is the case to be treated so that the size of the network represented by equation (1) is *N*. From consideration of realistic GRNs, we assume that the coupling parameters can be adjusted externally, which are effectively the set of control parameters. Specifically, for a GRN, we assume that the various coupling strengths among the nodes (genes) can be experimentally and systematically varied through application of specific targeted drugs. On a larger scale, the fate of a cell can be controlled by adding drugs to the cell-growth environment, which adjust the interaction parameters in the underlying network^28^. Although dynamical variables themselves can also be perturbed for the purpose of control, for GRNs this is unrealistic. For this reason, the scenario of perturbing dynamical variables will not be considered in this paper.

We focus on nonlinear dynamical networks with multiple coexisting attractors. For a given set of parameters *μ*, the multiple attractors (for example, stable steady states) and the corresponding basins are fixed. In the absence of stochasticity, for a given initial condition, the system will approach one of the attractors. Each attractor has specific biological significance, which can be regarded as either desired or undesired, depending on the particular function of interest. Suppose, without any control, the system is in an undesired attractor or is in its basin of attraction. The question is how to steer the system from the undesired state to a desired state by means of temporary and small parameter variations that are experimentally feasible.

### Control principle based on bifurcation

To motivate the development of a feasible control principle, we consider the simple case where the system is near a bifurcation point and control is to be applied to drive the system from one attractor to another through temporal perturbation to a single parameter. That is, the parameter variation is turned on and takes effect for a finite (typically short) duration of time. After control perturbation is withdrawn, the system is restored to its parameter setting before control but its state has been changed: the system will have moved to the basin of the desired attractor and will approach the desired attractor spontaneously. Let *μ*_0_ be the initial parameter value and the system is in an undesired attractor denoted as 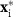, and let 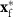 be the desired attractor to which the system is driven. Imposing control means that we change the parameter from *μ*_0_ to *μ*_1_. The dynamical mechanism to drive the system out of the initial attractor is bifurcations, for example, a saddle-node bifurcation at which the original attractor disappears and its basin is absorbed into that of an intermediate attractor^23^, denoted as 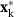. Turning on control to change *μ* from *μ*_0_ to *μ*_1_ thus makes the system approach 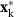. This process continues until the system falls into the original basin of 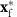, at which point the control parameter is reset to its original value *μ*_0_ so that the system will approach the desired attractor 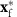. Success of control relies on the existence of a ‘path' from the initial attractor to the final one through a number of intermediate attractors. If a single parameter is unable to establish such a path, variations in multiple parameters can be considered, provided that such parameter adjustments are experimentally realizable. For a biological network, this can be achieved through application of a combined set of drugs^31^,^32^. However, even when potential complications induced by inter-drug interactions are neglected, the search space for suitable parameter perturbation can be prohibitively large if we allow all available parameters to be adjusted simultaneously. We demonstrate below that this challenge can be met by constructing an attractor network for the underlying system.

We note that not only is it now feasible to perturb gene expressions directly but also the technology for fine-tuning regulation strengths has become commonly available. For example, in our previous synthetic biology studies^29^,^33^, we used chemical inducers such as aTc, isopropyl-β-D-thiogalactoside and Arabinose to fine tune the strength of inhibition or activation. These chemical inducers do not change the protein abundance but rather chemically modify the protein structures upon binding so as to change their functions including the binding affinity. Other techniques such as RNA interference can also be used to fine-tune the gene regulation strength. In general, there are many different ways to tune gene regulation without chaining its transcription and translation.

### Attractor network for the T-cell signalling network

For a complex, nonlinear dynamical network, an attractor network can be constructed by defining each of all possible attractors of the system as a node. There exists a directed link from one node to another if an experimentally accessible parameter of the system can be adjusted to drive or control the system from the former to the latter. There can be multiple edges from one node to another, if there are multiple parameters, each enabling control. Starting from an initial attractor, one can identify, using all accessible parameters with variations in a physically reasonable range, a set of attractors that the system can be driven into. Repeating this procedure for all attractors in the system, we build up an attractor network that provides a blueprint for driving the whole networked system from any attractor to any other attractor in the system, assuming at the time that the latter attractor would lead to desired function of the system as a whole. As we demonstrate below, all these can be done using relatively small parameter perturbation in the sense that the modifications are small as compared with the ranges over which the corresponding parameters can vary.

**Figure 1:**
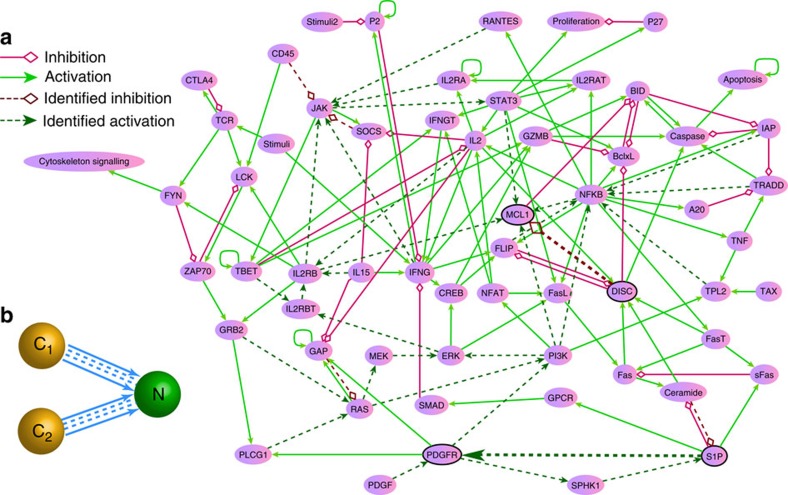
T-cell survival signalling network and its attractor network. (**a**) Structure of T-cell survival network: each node is labelled with its generic name, and the arrowhead and diamond-head edges represent activation and inhibition regulations, respectively. The inhibitory edges from ‘Apoptosis' to other nodes are not shown (for clarity). (**b**) Attractor network of the T-cell network, which contains three nodes: two cancerous states denoted as **C**_1_ and **C**_2_ and a normal state denoted as **N**. The two directed edges in the attractor network are multiple, each containing altogether 48 individual edges corresponding to controlling the 48 edges in the original network, which are indicated by the dark dashed lines, whereas the remaining edges in the original network are signified by the light solid lines. Our detailed computations reveal that parameter perturbation on any one of the 48 edges can drive the system from a cancerous state to the normal state. That is, regardless of whether the initial state is **C**_1_ or **C**_2_, with a proper modification to one of the 48 parameters, the system can be driven to the normal state **N**.

To demonstrate the construction of attractor networks, we take as an example a realistic biological network, T cells in large granular lymphocyte leukaemia associated with blood cancer. Specifically, apoptosis signalling of the T cells can be described by a network model: T-cell survival signalling network^34^,^35^, which has 60 nodes and 195 regulatory edges, as shown in Fig. 1a. Nodes in the network represent proteins and transcripts, and the edges correspond to either activation or inhibitory regulations. Experimentally, it was found that there are three attractors for this biophysically detailed network^34^,^35^. Among the three attractors, two correspond to two distinct cancerous states (denoted as **C**_1_ and **C**_2_) and one is associated with the normal state (denoted as **N**). The two cancerous states are biologically equivalent, differing only on node P2 (either activated or inactivated). As the T cells in large granular lymphocyte leukemia disease originate from the failure of the programmed T cells, the normal state corresponds to the situation where the node Apoptosis is activated while all other nodes are inactivated. By translating the Boolean rules into a continuous form using the method described in refs 36, 37 and setting the strength of each edge to unity, one can obtain a set of nonlinear differential equations for the entire network system. Direct simulation of the model indicates that there are three stable fixed point attractors, in agreement with the experimental observation^34^,^35^. The attractor network is thus quite simple: it has three nodes only, as shown in Fig. 1b. Testing all the 195 experimentally adjustable parameters, we find 48 edges from each cancerous attractor to the normal one (see Supplementary Table 1 for details). Our detailed computations reveal that parameter perturbation on any one of the 48 edges can drive the system from a cancerous state to the normal state. That is, regardless of whether the initial state is **C**_1_ or **C**_2_, with a proper modification to one of the 48 parameters, the system can be driven to the normal state **N**. We note that parameter perturbation does exist to drive the system from the normal state to a cancerous state (see Supplementary Note 1 for details).

### Control implementation based on attractor network

Given a nonlinear dynamical network in the real (physical) space, the underlying phase space dimension may be quite high, rendering analysis of the dynamical behaviours difficult. The attractor network is a coarse grained representation of the phase space, retaining information that is most relevant to the control task of driving the network system to a desired final state. Once an attractor network has been constructed, actual control can be carried out through temporary changes in a set of experimentally adjustable parameters. This should be contrasted to one existing approach^38^ that requires accurate adjustments in the state variables, which may not always be realistic.

**Figure 2:**
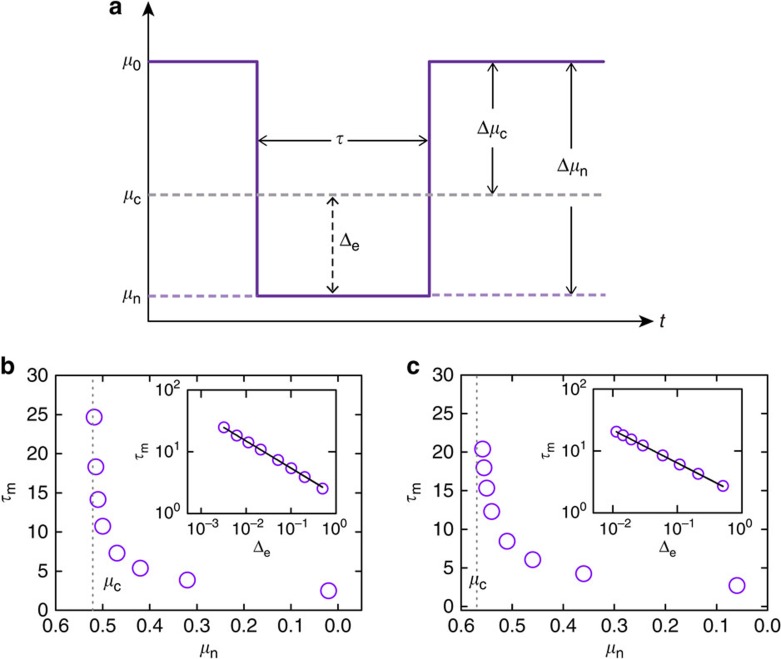
Relationship between edge control strength and minimal control time. For the T-cell network, (**a**) an inverted rectangular control signal of duration 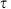 and amplitude 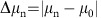, where *μ*_0_ is the original parameter value and *μ*_n_ is the control parameter value. A saddle-node bifurcation occurs for *μ*=*μ*_c_, so Δ_e_=*μ*_c_−*μ*_n_ is the excessive amount of the parameter change over the critical value *μ*_*c*_. (**b**,**c**) Minimal control time 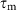 versus *μ*_n_, where parameter control is applied to the activation edge from node ‘S1P' to node ‘PDGFR' and to the inhibitory edge from ‘DISC' to ‘MCL1', respectively. These four nodes are indicated with the solid black circles in Fig. 1a. The corresponding plots on a logarithmic scale in the insets of (**b**,**c**) suggest a power-law scaling behaviour between 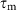 and Δ_e_ (equation (2)). The fitted power-law scaling exponents are *β*≈−0.44 and −0.55, respectively, for (**b**,**c**).

We detail how actual control can be implemented based on the attractor network for the T-cell system. To be concrete, we assume that the control signal has the shape of a rectangular pulse in the plot of a parameter versus time, as shown in Fig. 2a, where the control parameter is *μ* and the rectangular pulse has duration 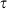 and amplitude 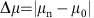, with *μ*_0_ denoting the nominal parameter value and *μ*_n_ being the value during the time interval when control is on. For the T-cell network, we set *μ*_0_=1.0. As *μ* is reduced, the system approaches a bifurcation point. (In other examples, a bifurcation can be reached by increasing a control parameter, as in the low-dimensional GRNs detailed in the Methods.) Extensive numerical simulations show that, to control the T-cell network from a cancerous state (**C**_1_ or **C**_2_) to the normal state **N**, there are wide ranges of choices for Δ*μ* and 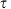. In fact, once *μ*_n_ is decreased through the bifurcation point *μ*_c_ at which the initial attractor loses its stability, the goal of control can be realized. The critical value *μ*_c_ for each parameter can be identified from the bifurcation analysis. In addition, for *μ*_n_<*μ*_c_, there exists a required minimum control time 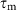, over which the system will move into the original basin of the target attractor before control is activated. Insofar as 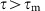, one does not need longer duration of control as the system will evolve into the target attractor following its natural dynamical evolution with the nominal parameter *μ*_0_. The value of 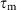 increases as *μ*_n_ is closer to *μ*_c_, where if *μ*_n_=*μ*_c_, 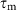 is infinite due to the critical slowing down at the bifurcation point *μ*_*c*_. Figure 2b,c show, respectively, for the T-cell network, the relationship between 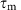 and *μ*_*n*_ when controlling the strength of the activation edge from the node ‘S1P' to the node ‘PDGFR', and that of the inhibitory edge from the node ‘DISC' to the node ‘MCL1' (cf., Fig. 1a, the nodes denoted as black circles and connected by bold coupling edges). The critical value *μ*_c_ (indicated by the dotted line) can be estimated accordingly. The insets in Fig. 2b and c show the corresponding plots of the relationships on a double logarithmic scale, with the horizontal axis to be Δ_e_=*μ*_c_−*μ*_n_, the *exceeded* value of *μ*_n_ over the critical point *μ*_c_. We observe the following power-law scaling behaviour:


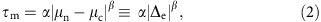


where *β* is the scaling exponent. The upper right region in the plane of the control parameters over the curve of 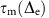, that is, the region with larger Δ_e_ value or longer duration 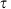, corresponds to the case where control is successful in the sense that the system can definitely be driven to the desired final state.

The power-law scaling relation for 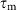 demonstrated in Fig. 2b,c for the T-cell network is quite general, as it also holds for two-node and three-node GRNs (see the Methods). For the T-cell system, the critical values of parameters for all the possible controllable edges from **C**_1_ or **C**_2_ to **N**, and the corresponding values of *α* and *β* in equation (2) are provided in Supplementary Table 1 and Supplementary Note 1. The control magnitude and time for some parameters are identical, for the reason that the logic relationship from the corresponding edges to the same node can be described as ‘AND' (c.f. Fig. 1) so that in the continuous-time differential equation model, all these in-edges are equivalent. (The control results for the two-node and three-node GRNs between any pair of nearest-neighbour attractors are listed in Supplementary Tables 2 and 3, respectively.)

Owing to the flexibility in choosing the control signal, our control scheme based on the attractor network is amenable to experimental implementation. We can also assign weights to the shortest paths in the attractor network. For example, if we assume all the links are equally implementable (Supplementary Table 1), the path from the **C** state to the **N** state involving any one of IL2RB, STAT3, NF-κB, PI3K or apoptosis to MCL1 has a relatively larger weight, representing the relatively more efficient control protocol as the required parameter change Δ_e_ can be minimized.

### Beneficial role of noise in control

More than three decades of intense research in nonlinear dynamical systems has led to great knowledge about the role of noise, in terms of phenomena such as stochastic resonance^39^,^40^, coherence resonance^41^,^42^, noise-induced chaos^16^ and noise-induced state transitions^43^. Common to all these phenomena is that a proper amount of noise can in fact be beneficial, for example, for optimizing the signal-to-noise ratio, for enhancing the signal regularity or temporal coherence, or for facilitating the transitions among the attractors. As our control mechanism is to make the system leave an undesired attractor and approach a desired one, noise in combination with parameter adjustments can facilitate the process of escaping from an attractor. To demonstrate this, we assume that the T-cell network is subject to Gaussian noise, which can be modelled by adding independent normal distribution terms **N**(0, *σ*^2^) to the system equations, where *σ* is the noise amplitude. We find that, with noise, the required magnitude of parameter change can be reduced. In fact, even when the controlled parameter *μ*_n_ has not yet reached the bifurcation point *μ*_c_, noise can lead to a finite probability for the system to escape the basin of the undesired attractor. We note that, in a recent work on stochastic control^43^, a method was presented to switch the dynamical states. In a real experimental setting, there can be different sources of noise such as temperature and metabolic burden. In our parametric control method, the control signals are flexible with adjustable duration and amplitude, and noise can enhance the flexibility.

**Figure 3:**
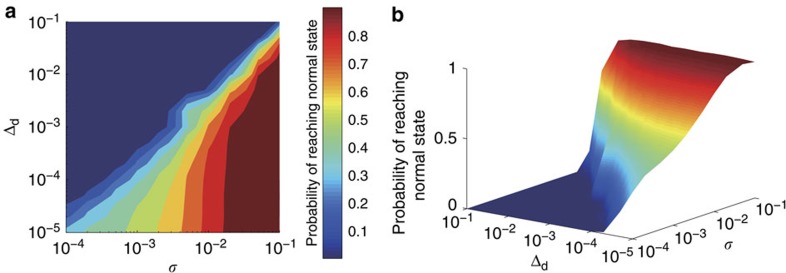
The benefit of noise in controlling the T-cell network. (**a**) Success rate to control the T-cell network from the cancerous state **C**_1_ to the normal state **N** using a combination of parameter perturbation and external noise (of amplitude) *σ*, where Δ_d_≡*μ*_n_−*μ*_c_ is the parameter deficiency. Warm colours indicate higher probability values of successful control. The perturbation duration is 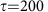. The results are averaged over 1,000 realizations. (**b**) A three-dimensional plot: success rate versus Δ_d_ and *σ*.

Suppose the control parameter is set to the value *μ*_n_, which is insufficient to induce escape from the undesired attractor in the absence of noise. When noise is present, the system dynamics is stochastic. To characterize the control performance, we carry out independent simulations starting from one cancerous state, for example, **C**_1_, but with insufficient control strength as characterized by the deficiency parameter Δ_d_≡*μ*_n_−*μ*_c_, and calculate the probability *P* of control success through the number of trials that the system can be successfully driven to the normal state **N**. Figure 3a shows, on a double logarithmic scale, the values of *P* in the parameter plane of *σ* and Δ_d_, where the control parameter is the strength of the activation edge from node ‘S1P' to node ‘PDGFR' in the T-cell network. A three-dimensional plot of *P* versus *σ* and Δ_d_ is shown in Fig. 3b. We see that, for fixed *σ*, *P* decreases with Δ_d_ but, for any fixed value of Δ_d_, the probability *P* increases with *σ*, indicating the beneficial role of noise in facilitating control. In the parameter plane, there exists a well-defined boundary, below which the control probability assumes large values but above which the probability is near zero. Testing alternative control parameters yields essentially the same results, because of the simplicity of the attractor network for the T-cell system and the multiple directed edges from each cancerous state to the normal state.

## Discussions

In nonlinear dynamics, controlling chaos has been studied for more than two decades^44^,^45^,^46^. Success, however, has been limited to low-dimensional dynamical systems with a very few positive Lyapunov exponents. Complex, nonlinear dynamical networks are generally high dimensional. Although mathematical controllability frameworks for such high-dimensional systems^1^,^2^ have been developed and extensively studied recently, the limitation is that the nodal dynamical processes must be assumed to be linear.

Controllability and actual control are two key issues associated with controlling nonlinear dynamics on complex networks. To assess the controllability, drastically different approaches than the linear controllability frameworks are needed. Although there were previous works on methods such as pinning control^12^,^47^ through alteration of the state variables, in realistic situations such strategies may be difficult to implement. Owing to the extremely diverse nonlinear dynamical behaviours that a network can generate, at the present there is no universal framework for actual control of complex networks with nonlinear dynamics through realistic perturbation. The mathematical control theory for linear dynamical systems aims to control the detailed states of all of the variables, which is in fact an overkill for most systems. For nonlinear dynamical networks, a physically meaningful approach may not require detailed control of all state variables. With this relaxation of the control requirement, it may be possible to develop a framework of controllability and devise actual control strategies for nonlinear dynamical networks based on physical/experimental considerations. In particular, a common feature of nonlinear dynamical systems is the emergence of distinct, coexisting attractors^16^,^48^. Often the performance and functions of the system are determined by the particular attractor that the system has settled into, to which the detailed states of the dynamical variables are not relevant. The key is thus to develop control principles whereby we nudge a complex, nonlinear system from attractor to attractor through small perturbation to a set of physically or experimentally feasible parameters. The main message of this paper is that a controllability framework can be developed for nonlinear dynamical networks based on controlling attractors.

Assuming that the networked system will evolve into an undesired attractor, the control goal is to apply perturbation to steer the system into a desired attractor. This can be accomplished by identifying a final attractor leading to the desired performance and choosing a set of experimentally adjustable parameters. If the perturbation can drive the system from the undesired state to the desired attractor, there exists a control path between the former and the latter, regardless of whether there are intermediate attractors on the path. Suppose we have found all the attractors of the system (see Supplementary Note 2 for a systematic method). In terms of some specific performance criteria, we can classify the attractors as undesired, desired or intermediate. If there is a control path from any undesired attractor to the desired attractor, the networked system is deemed controllable. An attractor network can then be constructed, with nodes and edges being the attractors and control paths, respectively. The topology and properties of the attractor network, such as the network diameter and the total amount of the parameter perturbation, effectively quantify the controllability of the original network. We demonstrate our idea of control and construction of attractor networks using realistic networks from systems and synthetic biology. We also find that noise can facilitate control of nonlinear dynamical networks, and we provide a physical understanding of this phenomenon (Methods).

Our framework can be adopted to controlling nonlinear dynamical networks other than the GRNs. For example, for the Northern European power grid network recently studied^49^, a rewiring method was proposed and demonstrated to be able to enhance the system stability through the addition of extra transmission lines. For a power grid network, the synchronous states are desired, whereas other states, for example, limit cycles, are detrimental. Treating the link density (or number) as a tunable parameter, the minimum transfer capacity required for extra lines to realize the control can be estimated by our method. Another example is Boolean networks with discrete dynamics, for which a perturbation method was proposed based on modification of the update rules to rescue the system from the undesired states^50^. In terms of our method, an attractor network can be constructed based on perturbation to multiple parameters to drive the system out of the undesired, damaged states towards a normal (desired) state. For biological systems, an epigenetic state network (ESN) approach was proposed^51^ to analyse the transitions among different phenotypic processes. In an ESN, nodes represent attractors and edges represent pathways between a pair of attractors. By construction, different parameter values would result in a different ESN. (See Supplementary Note 3 and Supplementary Fig. 2 for an example of using the principle of attractor network to control a stochastic, biochemical reaction network.) In our attractor network, nodes are attractors but edges are directed and represent controllable paths (through parameter perturbation) to drive the system from one attractor to another.

At the present, it is difficult to formulate a rigorous mathematical controllability framework for nonlinear dynamical networks. A challenge is that different parameter perturbation will typically lead to a different attractor network. Moreover, as the attractor network is directed and typically does not have an all-to-all coupling configuration, multiple parameter perturbation may be needed to realize control (for example, as demonstrated using a three-node GRN system - see the Methods), rendering the amount of computation prohibitive for relatively large dynamical networks. Successfully addressing these issues will ultimately enable us to achieve the grand goal of controlling nonlinear dynamical networks.

## Methods

### Construction of attractor networks

For a system with known attractor basins and given initial and final states, the attractor network can be constructed through the following steps.

1. Find all the parameters that can be adjusted externally and determine the possible range of variation of each tunable parameter based on experimental considerations and computational efficiency. For example, the T-cell network has 195 tunable parameters. Using the differential equation model one can test each parameter separately to determine its range of variation that can drive the system from one attractor to another. (Testing different parameter combinations is computationally prohibitive.)
2. For a tunable parameter, choose a small set of values in its variable range, which include its original or nominal value.
3. Simulate the system dynamics for each parameter value, starting from the initial attractor and determine the final attractor. Record the parameter values that can drive the system into the basin of the desired attractor. For example, for the T-cell network, parameter variations lead to saddle-node bifurcations, rendering applicable a straightforward bisecting algorithm to reduce the computational complexity in searching for the set of parameter values that result in a desired attractor.
4. An attractor network can be constructed after all tunable parameters have been tested, from which distinct control paths can be identified for any given pair of initial and final attractors. (For the systems treated in this paper, the computational time required to construct the attractor network is listed in Supplementary Table 4.)

For systems whose attractors are unknown *a priori*, it is necessary to execute an attractor finding algorithm before an attractor network can be reconstructed. An example of such an algorithm is presented in Supplementary Note 2. The issues of multiple parameter-based control and computational cost are discussed in Supplementary Notes 4 and 5, respectively.

### Bifurcation diagram

Bifurcation diagram represents a visualization method to characterize a system's steady-state behaviour with respect to parameter variation. In this paper, we used the MATCONT package in MATLAB to draw the bifurcation diagrams^52^. The bifurcation curves in MATCONT are computed based on the numerical continuation algorithm with a predictor-corrector procedure to enhance the stability and reduce the computational time. For the two-node GRN example below, we calculate the bifurcation diagrams with respect to all four tunable parameters, based on which the attractor network can be constructed.

### A two-node GRN

In spite of the simplicity of its attractor network, the original T-cell network itself is still quite complicated from the point of view of nonlinear dynamical analysis. To have a better understanding of our control mechanism, we study GRNs of relatively low dimensions and carry out a detailed analysis of the associated attractor networks.

**Figure 4:**
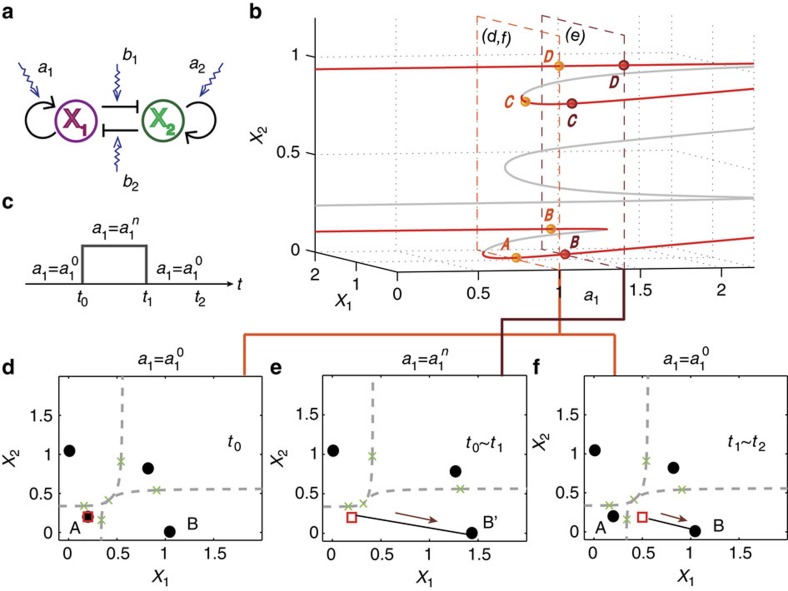
Control of a GRN of two nodes. (**a**) Simplified model of the two-node GRN, where the arrowhead and bar-head edges represent activation and inhibition regulations, respectively, and the sawtooth lines denote the strength of the tunable edge. (**b**) Bifurcation diagram with respect to the control parameter *a*_1_, where the red and grey solid lines denote the stable and unstable steady states, respectively. In the two parallel cross-sections (with dashed line boundaries) for 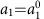 and 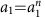, the yellow and brown dots represent the corresponding stable attractors, respectively. (**c**) Control signals required to drive the system from attractor **A** to attractor **B**. In **d**–**f**, grey dashed lines represent the basin boundaries; black solid circles and green crosses denote attractors and unstable steady states, respectively. (**d**) For the initial parameter setting, 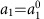, the system is at a low concentration state **A**, and the target state is **B**. (**e**) By changing *a*_1_ from 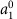 to 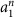, attractor **A** is destabilized and its original basin is absorbed into that of the intermediate attractor **B**′, so the system approaches **B**′. (**f**) When control perturbation upon *a*_1_ is released, the landscape recovers to that in **d**. Once the system has entered the basin of the target state **B** during the process in **e** it will evolve spontaneously towards **B**. Parameters in simulation are 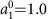, 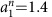, *t*_0_=0, *t*_1_=23 and *t*_2_=40.

We use a two-node GRN to understand the dynamical underpinning of the attractor network. As shown in Fig. 4a, the network contains two auto-activation nodes (genes) and together they form a mutual inhibitory circuit. Such a topology was shown to be responsible for the regulation of blood stem cell differentiation^53^. In addition, it is conceivable that such topologies can be constructed with tunable inputs using synthetic biology approaches^29^.

In a typical experimental setting, four coupling parameters can be adjusted externally through the application of repressive or inductive drugs. To demonstrate attractor network and control implementation, we consider the parameter regime in which the system has four stable steady states (attractors) that correspond to four different cell states during cell development and differentiation. In particular, the dynamical network can be mathematically described as,


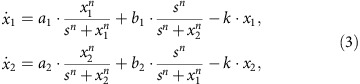


where the dynamical variables (*x*_1_, *x*_2_) characterize the protein abundances of the gene products, *k* denotes the degradation rate of each gene, and the tunable parameters *a*_1_, *a*_2_, *b*_1_ and *b*_2_ represent the strengths of auto or mutual regulations. In a GRN, the dynamical behaviours of inhibition and activation are captured by the Hill function: *f*(*x*)=*x*^*n*^/(*x*^*n*^+*s*^*n*^) for activation and *f*(*x*)=*s*^*n*^/(*x*^*n*^+*s*^*n*^) for inhibition, where the parameter *s* characterizes half activation (or inhibition) concentration (for *x*=*s*, the output is 0.5), and *n* quantifies the correlation between the input and output concentrations, with a larger value of *n* corresponding to a stronger inhibition or activation effect. For any specific GRN, the values of both *s* and *n* can be determined experimentally. For simplicity, we assume the system to be symmetric in that inhibition and activation share the same Hill function (that is, with the same parameters *s* and *n*). To have four attractors, we set the auto activation strengths, *a*_1_ and *a*_2_, to 1.0, and mutual inhibition strengths, *b*_1_ and *b*_2_, to 0.2. The value of the degradation rate *k* is set to 1.1, taking into account the effects of protein degradation and cell volume expansion.

Figure 4b–f shows a particular process of controlling the system from an initial state, denoted as **A**, in which both *x*_1_ and *x*_2_ have low abundance, to a final state **B** where *x*_1_ and *x*_2_ have high and low abundance, respectively. From the bifurcation diagram (Fig. 4b) with respect to the control parameter *a*_1_, we see that, as *a*_1_ is increased from 1.0 to 1.4, in the lower branch the initial attractor **A** is destabilized through a saddle-node bifurcation. The control signal is shown in Fig. 4c, where the original and perturbed parameter values are denoted as 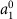 and 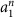, respectively. The bifurcation-based control process is shown in Fig. 4d–f, where Fig 4d exhibits the phase space of the system before control (*a*_1_=1.0). When control is activated so that *a*_1_ is set to *a*_1_=1.4, the original basin of attractor **A** merges into the basin of an intermediate attractor **B**′, and the system originally in **A** starts to migrate towards the intermediate attractor **B**′, as indicated by the arrowed trajectory in Fig. 4e. Control perturbation upon *a*_1_ can be withdrawn once the system enters the region belonging to the original basin of the target attractor **B**, after which the system spontaneously evolves into **B** for *a*_1_=1.0, as shown in Fig. 4f.

**Figure 5:**
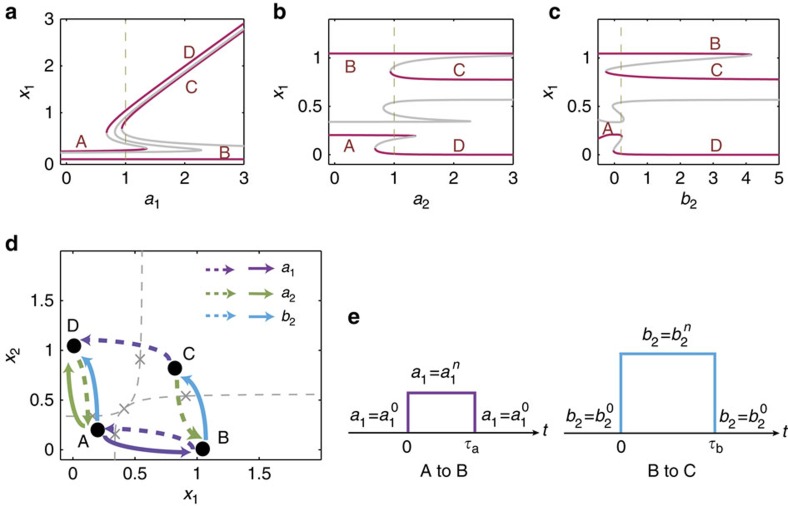
Attractor network construction for a GRN of two nodes. (**a**–**c**) Bifurcation diagrams with respect to the coupling parameters *a*_1_, *a*_2_ and *b*_2_, respectively, where each bifurcation point can be exploited to design control. (**d**) The corresponding attractor network, in which a directed edge corresponds to an elementary control that is designed to steer the system from the original attractor to the directed one. The solid and dashed edges, respectively, denote the positive and negative changes in the corresponding control parameters. (**e**) Sequential control signals required to drive the system from attractor **A** to attractor **C** through the path **A**→**B**→**C**. In simulation, the original parameter values are 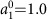 and 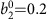. We set 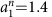, followed by setting 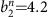, and the respective durations of the parameter perturbation are 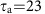 and 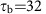.

To obtain a global picture of all possible control outcomes, we construct the attractor network for the two-node GRN system, assuming that three parameters: *a*_1_, *a*_2_ and *b*_2_, are available for control. The corresponding bifurcation diagrams are shown in Fig. 5a–c, from which all saddle-node bifurcations can be identified for control design. When all the attractors are connected with directed and weighted edges through the control processes, that is, when none of the attractor is isolated, we obtain an attractor network, as shown in Fig. 5d. Specifically, the edge weight can be assigned by taking into account the key characteristics of control such as the critical parameter strength *μ*_c_ and the power-law scaling behaviour of the required minimum control time 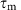 (see Supplementary Table 2 for details). From the attractor network, we can find all possible control paths for any given pair of original and desired states.

From Fig. 5d, we see that the two-node GRN system is fully controllable as any of the coexisting attractors is reachable through proper sequential controls to the available parameters. The concept of attractor network is appealing because it provides a clear control scenario to drive the system from any initial attractor to any desired attractor. In fact, the attractor network provides a blueprint that can be used to design a proper combination of parameter changes to induce the so-called synergistic or antagonistic effects^54^. For example, **A** is not directly connected with **C**, neither is **B** directly connected to **D**. However, the system can be steered from **A** to **B** through perturbation on *a*_1_, and then from **B** to **C** through a change in *b*_2_, as shown in Fig. 5e. Another example to demonstrate the need of multiple parameter perturbation is to control the system from **B** to **D**. A viable control path is **B**→**C**→**D**, which can be realized through perturbation on parameters (*b*_2_, *a*_1_). We also see that the two **B**→**A**→**D** paths can be realized through parameter changes in (*a*_1_, *a*_2_) and (*a*_1_, *b*_2_), respectively. A phase diagram illustrating how different choices of the parameters affect the final attractor is provided in Supplementary Note 6 and Supplementary Fig. 1.

When multiple control paths exist from an initial attractor to a final one, a practical issue is to identify the optimal path that is cost effective and robust. The concept of weighted-shortest path can be used to address this issue. Particularly, the weights of edges can be determined from experimental considerations such as the cost, limitation in drug dose, the control duration time and so on.

### Beneficial role of noise in nonlinear control

The role of noise in facilitating control of a nonlinear dynamical network can be understood using the concept of potential landscape^55^,^56^,^57^ or Waddington landscape^58^ in systems biology, which essentially determines the biological paths for cell development and differentiation^59^,^60^. The potential landscape was used to manipulate time scales to control stochastic and induced switching in biophysical networks^43^,^60^. Intuitively, the power of the landscape concept can be understood by resorting to the elementary physical picture of a ball moving in a valley under gravity. The valley corresponds to one stable attractor. To the right of the valley there is a hill, or a potential barrier in the language of classical mechanics. The downhill side to the right of the barrier corresponds to a different attractor. Suppose the confinement of ball's motion within the valley is undesired and one wishes to push the ball over the barrier to the right attractor (desired). If the barrier is high, there will be little probability for the ball to move across the top of the barrier towards the desired attractor. In this case, a small amount of noise is unable to enhance the crossover probability. However, if the barrier height is small, a small amount of noise can push the ball over to desired attractor on the right side of the barrier. Thus, the beneficial role of noise is more pronounced for small height of the potential barrier, a behaviour that we observe when controlling the T-cell network (Fig. 3). In mechanics, the system can be formulated using a potential function so that, mathematically, the motion of the ball can be described by the Langevin equation, which has been a paradigmatic model to understand nonlinear phenomena such as stochastic resonance^39^,^61^,^62^. In the past few years, a quantitative approach has been developed to map out the potential landscape for gene circuits or GRNs^55^,^63^,^64^. In nonlinear dynamical systems, a similar concept exists—quasipotential^65^,^66^,^67^, which plays an important role in understanding phenomena such as noise-induced chaos.

For an attractor network, in the presence of noise, each node corresponds to a potential valley of certain depth that characterizes the stability of the attractor. For a fixed depth, noise of larger amplitude *σ* leads a higher escaping probability or shorter escaping time. When the amplitude of the control signal is not sufficient to drive the system across the local potential barrier, noise can facilitate control by pushing the system out of the undesired valley (attractor).

**Figure 6:**
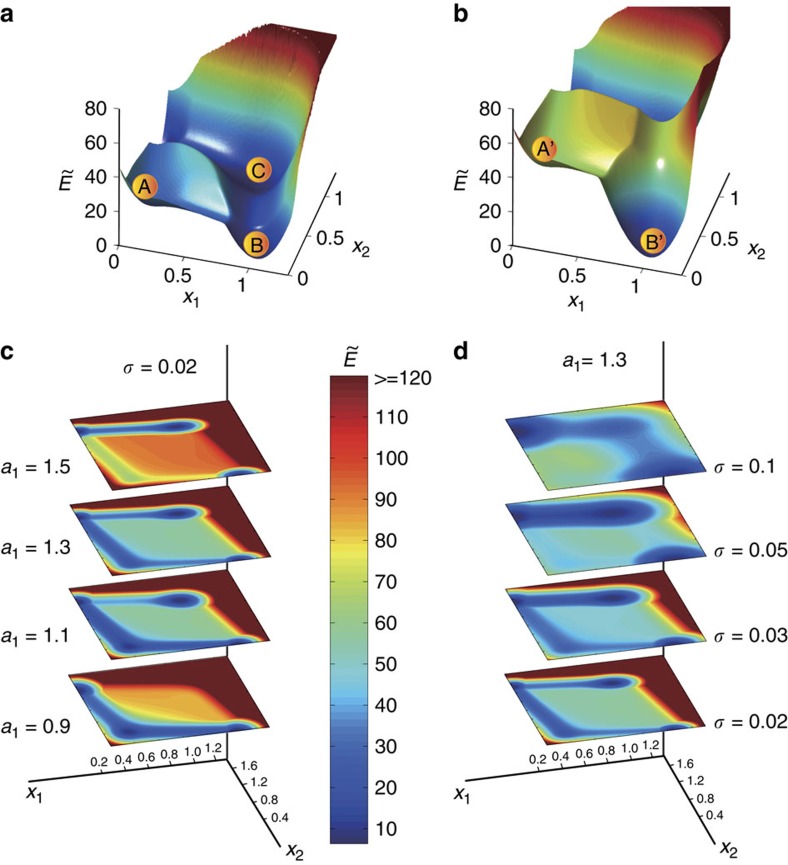
Illustration of pseudo potential landscape. ‘Pseudo' potential 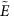 of the two-node GRN system (**a**) for *a*_1_=1.0 (Δ_d_≈0.3549), *σ*=0.05 and (**b**) for *a*_1_=1.3 (Δ_d_≈0.0549), *σ*=0.05. Regions of warm and cold colours indicate the states with large and small pseudo energies, respectively. (**c**) For fixed *σ*=0.02, two-dimensional representation of 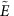 for a number of values of *a*_1_. (**d**) For fixed *a*_1_=1.3, two-dimensional representation of 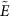 for a number of values of *σ*.

The potential landscape for a GRN under Gaussian noise can be constructed from the dynamical equations of the system using the concept of ‘pseudo' energy^57^. For the two-node GRN system (equation (3)) subject to stochastic disturbance *N*(0, *σ*^2^), we can compute the potential landscape for any combination of some system parameter (say *a*_1_) and the noise amplitude *σ*. Figure 6a,b shows two examples of the landscape (in three-dimensional representation) for *a*_1_=1.0 and *a*_1_=1.3, where the noise amplitude is *σ*=0.05. We see that, for example, for *a*_1_=1.0, there are four valleys (attractors). Figure 6c shows, for *σ*=0.02, a two-dimensional representation of the pseudo-energy for a number of values of *a*_1_. We observe that, for a same noise amplitude, as *a*_1_ is increased, the transition rate from **A** to **B** becomes higher (the colour becomes warmer).

What if the system is at an attractor that is deep in its basin and not close to the boundary? As our approach is based on parameter adjustment, such a case can still be effectively controlled in the sense that the system can be brought out of the attractor. For example, consider the attractor **C** for *a*_1_=1.5 in the two-node GRN system, which is deep inside its own basin, as shown in Fig. 6c. When parameter perturbation is applied to the system, its energy landscape and basin structure are changed (from top to bottom). Under control, the attractor ‘moves' towards the basin boundary and is destroyed when it reaches the boundary, thereby bringing the system out of this attractor. Another example is attractor **A** in Fig. 6c, where it can be seen that, when the control parameter *a*_1_ increases its value from 0.9 to 1.5, its basin changes and the attractor moves towards the boundary as well. Similar plots but for fixed *a*_1_=1.3 and different values of *σ* are shown in Fig. 6d. For *a*_1_=1.3, the pseudo energy for **A** (the original valley at the lower-left corner) becomes higher, and the path for the transition from **A** to **B** becomes more pronounced. Further increasing *a*_1_ towards the critical value (about 1.35) raises the energy of **A** to the level of the potential barrier, effectively eliminating the corresponding valley and the attractor itself. Note that, for a fixed value of *a*_1_, increasing noise amplitude can lead to a mixture of cold colours, meaning that the valley range becomes wider and the ridge between two adjacent valleys becomes shallower, resulting in a higher transition probability for each attractor.

### A three-node GRN

**Figure 7:**
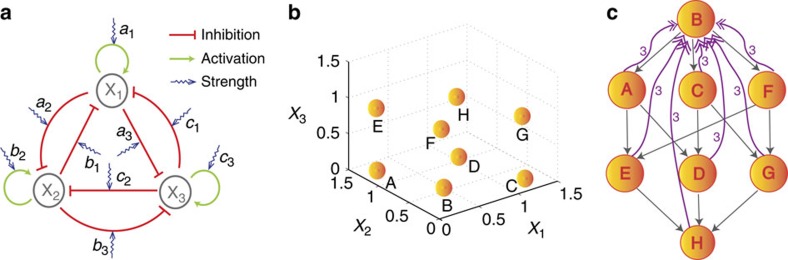
A GRN of three nodes and its attractor network. (**a**) Schematic illustration of a three-node GRN system. The arrowhead and bar-head edges represent activation and inhibitory regulations, respectively. The sawtooth lines specify that the corresponding edge strength is experimentally adjustable. (**b**) Coexisting attractors (**A** to **H**) in the phase space. (**c**) The underlying attractor network, where each node represents an attractor and each weighted directed link indicates that its strength can be experimentally tuned to steer the system from the starting attractor to the pointed attractor. Each grey directed link with a single arrow indicates that only one parameter is needed to achieve control, and each purple link with a double-arrow and the label 3 represent the case where three parameters are required to achieve control.

We also study a three-node GRN system, as shown in Fig. 7a. Similar to the two-node GRN system, there exist both auto and mutual regulations among the nodes. All the interactions are assumed to be characterized by the same parameters, *s* and *n*, in the Hill function. The nonlinear dynamical equations of the system are^26^,^68^:


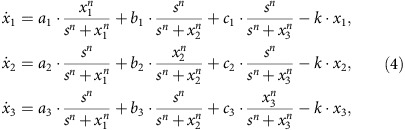


where the state variables (*x*_1_, *x*_2_ and *x*_3_) represent the abundances of the three gene products, the auto-activation parameters *a*_1_, *b*_2_, *c*_3_ and the mutual-inhibition parameters *a*_2_, *a*_3_, *b*_1_, *b*_3_, *c*_1_, *c*_2_ are all experimentally accessible. To be concrete, initially all the auto activation and mutual inhibition parameters are set to be 1.0 and 0.1, respectively, and *k* (the degradation rate) can be conveniently set to unity. The parameters in the Hill function are *n*=4 and *s*=0.5. There are altogether eight attractors in this system, as shown in Fig. 7b, which are distributed symmetrically in the three-dimensional state space. For example, attractor **H** has relatively high values for all three dynamical variables, and attractor **B** exhibits the opposite case with low abundances. For attractors **A**, **C** and **F**, one of the three state variables is high and the other two are low. For attractors **D**, **E** and **G**, one of the three state variables is low but the other two are high.

From numerical simulations, we find that the features of control are essentially the same as those for the two-node GRN system, in terms of characteristics such as the existence of critical control strength and the power-law scaling behaviour of the minimum control time (see Supplementary Table 3 and Supplementary Notes 7 and 8 for details). We construct the attractor network in Fig. 7c through combinations of all eight attractors (as nodes) and directed elementary controls (as weighted directed edges). Information in Supplementary Table 3 can also be used to estimate the respective weights of the edges. From the attractor network, for any given pair of initial and final states, we can identify all the viable control paths. Furthermore, the weighted-shortest path can be calculated once the edge weights are determined.

We note that, typically, the attractor network based on elementary control is not an all-to-all directed network so that certain control paths are absent, for example, from attractor **H** to **B**. Therefore, we need to extend the control method to a combination of multiple parameters. From Fig. 7c, we note that attractor **H** has incoming links only, whereas **B** has outgoing links only. The structure of the attractor network thus indicates that full control can be achieved (that is, to drive the system from any initial attractor to any final attractor) if we can build a directed path from **H** to **B**. Since attractors **B** and **H** have low and high abundance, respectively, in the phase space, we modify the three auto-activation parameters simultaneously to steel the system from **H** to **B**. We find that the relationship between control strength and minimal control time also follows the power law scaling with the scaling parameters *μ*_*c*_≈0.7973, *α*≈1.26 and *β*≈−0.77. In fact, when all the auto-activation strengths are smaller than *μ*_*c*_, the system has one attractor only: **B**, meaning that perturbation based on parameter combinations can be used to realize the control from other attractors as well, that is, from one of the attractors **A**, **C**, **D**, **E**, **F** and **G** to attractor **B**.

### Pseudo potential landscape

For a dissipative, nonlinear dynamical system subject to noise, we can construct a pseudo potential landscape based on the state probability distribution. Assume that, asymptotically, the system approaches a stationary distribution. For a canonical dynamical system, the potential can be defined as *E*(**x**)=−log*P*(**x**), where *P*(**x**) is the probability density function. For a conservative dynamical system, the direction of system evolution is nothing but the direction of the gradient of the potential function. However, this does not hold for dissipative dynamical systems. The potential function thus does not have the same physical meaning as that for a conservative system, henceforth, the term pseudo potential. This approach can be adopted to GRNs.

To obtain the stationary distribution, we use the modified weighted-ensemble algorithm^57^, which offers faster convergence than, for example, the traditional random walk method. To be illustrative, we take the two-node GRN system (equation (3)) as an example to demonstrate how the pseudo potential landscape can be numerically constructed. The state space of the two-dimensional dynamical system is partitioned into an *M* × *M* lattice with reflective boundaries conditions. Initially, the probability *P*_*m*,*n*_(*t*) of all gird points are set to be uniform. The simulation time is divided into *T* steps, where each step has the duration Δ*t*. At the beginning of each step *t*, there are *N* walkers randomly distributed at the grid point (*m*, *n*), which carry equal weight *P*_*m*,*n*_(*t*)/*N* and perform random walk under the system dynamics and noise. The locations of these walkers in the grid are recorded at the end of each time step, and the probability at the next time step, *P*_*m*,*n*_(*t*+1), is the summation of the probabilities carried by all the walkers at time *t*. At time (*t*+1), *N* new walkers carrying the updated probability at each grid point perform random walk again on the grid. This procedure repeats until the probability distribution becomes stationary, say *P*_*m*,*n*_, which gives the pseudo potential landscape as 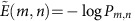. Numerically, the time evolution of all walkers can be simulated using the second-order Heun method for integrating stochastic differential equations. For Fig. 6, the state space is divided into a 500 × 500 grid. At each grid point, there are *N*=20 walkers, each evolving *T*=2,000 time steps with Δ*t*=10^−4^.

Instead of calculating the stationary probability distribution density, an alternative approach to constructing the potential landscape is the Freidlin and Wentzel's large deviation theory^69^. According to this theory, first, one maps the stochastic dissipative system of interest to a Hamiltonian system^70^. One then estimates the transition rate from an attractor to a saddle point as *r*∼exp(−*S*_0_/*σ*), where *σ* is the noise amplitude and *S*_0_(·) characterizes the action functional associated with the optimal energy path, which can be numerically solved through an unconstrained nonlinear optimization method^51^,^55^. As this theory relies on solving a nonlinear optimization problem, in order to visualize the energy landscape, it is necessary to use a large number of state values **x** and solve the optimization problem for each, which is computationally exhaustive. We find that, to capture the essential features of the energy landscape, it suffices to use the stationary distribution method.

In the weak noise limit *σ*→0, according to the theory, to the leading order, the transition rate *r* from one fixed point to a nearby saddle point **x**_sp_ can be approximated as *r*∼exp[(−*S*_0_/(**x**_sp_)]. The minimum control time satisfies 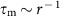, so we have 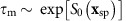. From equation (2), we obtain


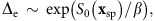


indicating that the exceeded value Δ_e_ of the control parameter *μ* is exponentially related to the optimal energy *S*_0_ associated with the path between the initial state and the nearby saddle point in the potential landscape.

## Additional information

**How to cite this article:** Wang, L.-Z. *et al*. A geometrical approach to control and controllability of nonlinear dynamical networks. *Nat. Commun.* 7:11323 doi: 10.1038/ncomms11323 (2016).

## Supplementary Material

Supplementary Information Supplementary Figures 1-2, Supplementary Tables 1-4, Supplementary Notes 1-8 and Supplementary References
