# Supplementary material for: A geometrical approach to control and controllability of nonlinear dynamical networks
Source: Nat Commun. 2016 Apr 14;7:11323. doi: 10.1038/ncomms11323 (PMC4834635; doi:10.1038/ncomms11323)
Supplement: Supplementary Information — Supplementary Figures 1-2, Supplementary Tables 1-4, Supplementary Notes 1-8 and Supplementary References [file ncomms11323-s1.pdf]

Supplementary Figures

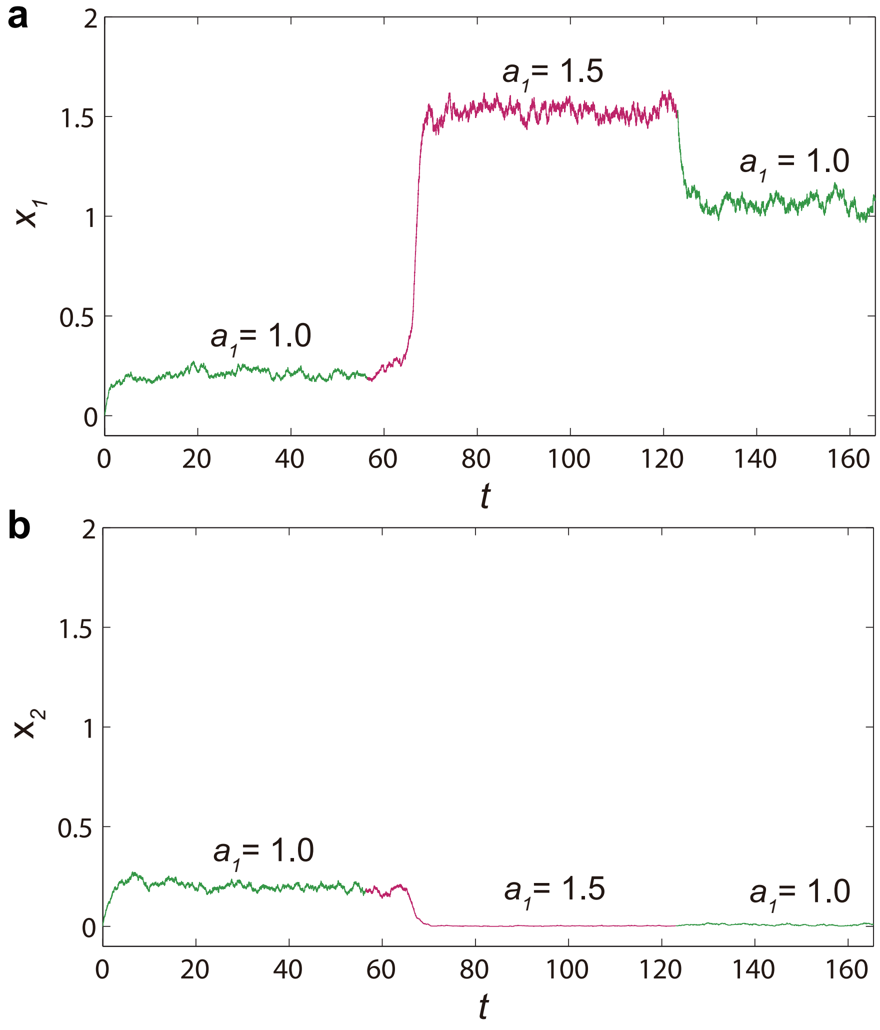

Supplementary Figure 1: **Control of a stochastic model.** Time series of variables x_1 (a) and x_2 (b). In the simulation, we multiply the values a_1, a_2, b_1, b_2 and s by 100 to obtain biologically reasonable protein abundances, while other parameters are unchanged. The final stochastic results are rescaled to match the ODE model results. Green and red lines represent the systems with $a_1 = 1.0$ and $a_1 = 1.5$, respectively.

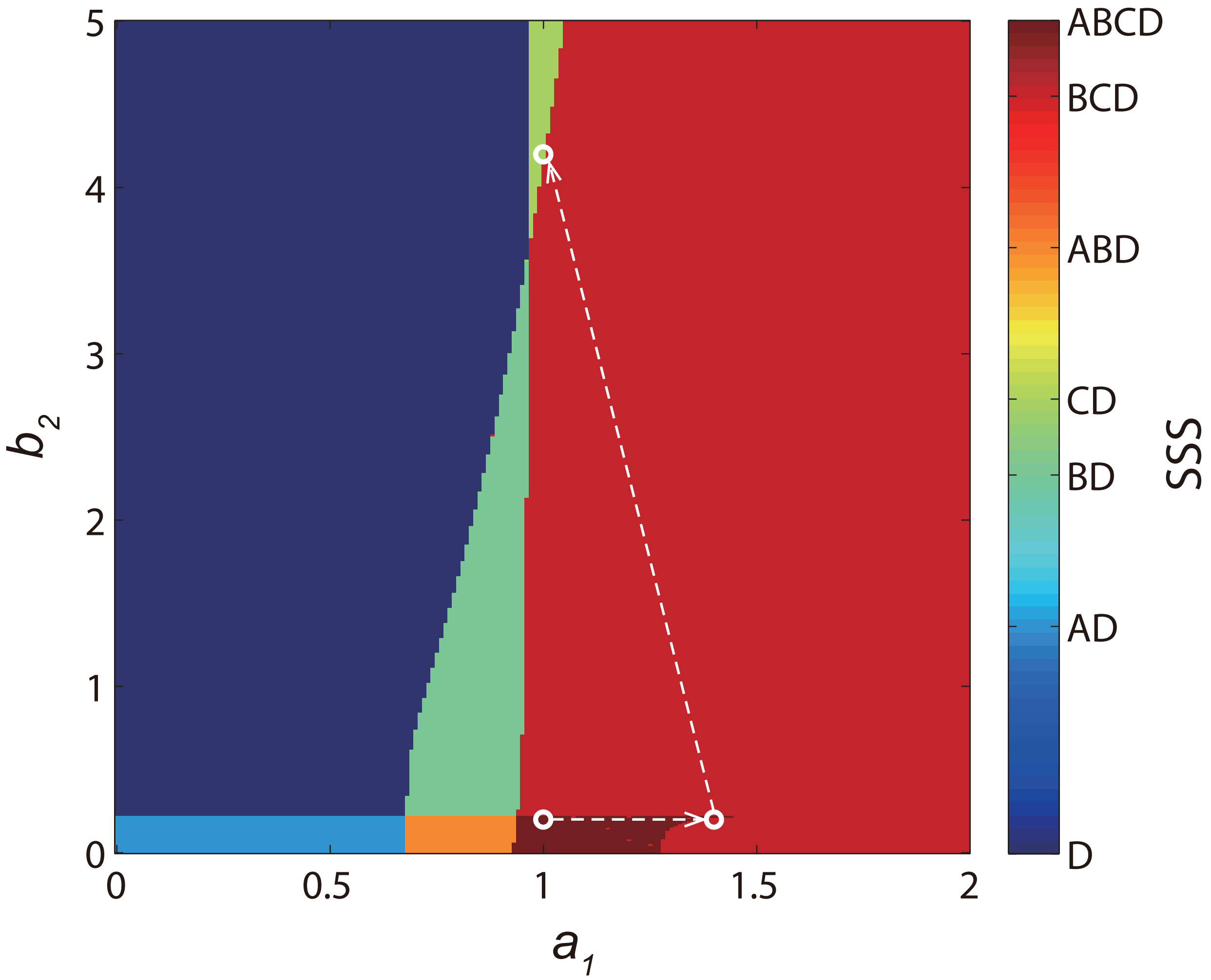

Supplementary Figure 2: **Phase Diagram of two-node GRN system.** For the two-node GRN system treated in the main text, final state of the system in the parameter plane (a_1, b_2), where each colored region represents a specific combination of attractors. Other parameters are set as $a_2 = 1.0$ and $b_1 = 0.2$. Each white circle represents a whole set of possible attractors in one system and the white dash lines illustrate the control procedure. The four-attractor state $\mathbf{ABCD}$ can be identified toward the bottom of the phase diagram.

Supplementary Tables

Supplementary Table 1: **Edge control in the T-cell system.** The first and the second columns, respectively, give the executor and the receiver of the coupling edges in the T-cell system. For control associated with each edge, the minimum control time τ_{m} exhibits a power-law scaling behavior: $\tau_{\mathrm{m}} = \alpha \cdot |\mu_{\mathrm{c}} - \mu_{\mathrm{n}}|^{\beta}$, where μ_{c} is the critical coupling strength (the third column). The fourth and the fifth columns list the scaling parameters α and β obtained by least squares fitting, with mean squared error (MSE) given in the sixth column. Note that Apoptosis represents the outcome of cellular signaling and it has inhibitory regulations to all the other nodes in the network. If Apoptosis is activated, the system will reach the desired normal state. Because of this the node Apoptosis appears in every row of the first column.

From	To	Critical coupling strength μ_c	α	β	MSE
S1P or PDGF or Apoptosis	PDGFR	0.5210	1.9921	−0.4360	0.0070
GRB2 or PDGFR or GAP or Apoptosis	RAS	0.5329	1.5664	−0.4892	0.0052
IL2RA or IL2RB or RANRES or IFENG or SOCS or CD45 or Apoptosis	JAK	0.5408	1.5619	−0.5154	0.0272
PDGFR or Apoptosis	SPHK1	0.5496	1.7948	−0.4904	0.0001
SPHK1 or Ceramide or Apoptosis	S1P	0.5697	1.7324	−0.5509	0.0008
RAS or Apoptosis	MEK	0.5805	1.5169	−0.5009	0.0111
JAK or Apoptosis	STAT3	0.5935	1.7482	−0.4587	0.0536
TPL2 or PI3K or FLIP or TRADD or IAP or Apoptosis	NFkB	0.6060	1.2286	−0.6366	0.0004
FDGFR or RAS or Apoptosis	PI3K	0.6187	1.3917	−0.5764	0.0786
MEK or PI3K or Apoptosis	ERK	0.6225	1.7762	−0.4363	0.0500
ERK or TBET or Apoptosis	IL2RBT	0.6412	1.7303	−0.4581	0.0285
IL2RBT or IL2 or IL15 or Apoptosis	IL2RB	0.6520	1.6082	−0.5078	0.0980
DISC or IL2RB or STAT3 or NFkB or PI3K or Apoptosis	MCL1	0.6596	1.5745	−0.5480	0.0510

Supplementary Table 2: **Controlling a two-node GRN system.** For the two-node GRN system discussed in the main text, the underlying attractor network consists of four nodes, denoted as $\mathbf{A}$, $\mathbf{B}$, $\mathbf{C}$, and $\mathbf{D}$, respectively. The first column represents all possible elementary controls among the nodes in the attractor network. The second column shows the specific coupling parameter adjusted to realize the elementary control and the corresponding critical coupling strength. The remaining columns show the parameters α and β as well as the fitting error (MSE) in the power-law scaling of the minimum control time τ_{m}.

Elementary control	Critical coupling strength μ_c	α	β	MSE
$\mathbf{A}$ to $\mathbf{B}$	$a_1 = 1.3523$	2.7590	-0.6688	0.2148
$\mathbf{A}$ to $\mathbf{B}$	$b_1 = 0.2206$	0.1735	-0.9085	0.0014
$\mathbf{A}$ to $\mathbf{D}$	$a_2 = 1.3523$	2.7590	-0.6688	0.2148
$\mathbf{A}$ to $\mathbf{D}$	$b_2 = 0.2206$	0.1735	-0.9085	0.0014
$\mathbf{B}$ to $\mathbf{A}$	$a_1 = 0.6800$	2.0415	-0.5539	0.0990
$\mathbf{B}$ to $\mathbf{C}$	$b_2 = 4.1557$	5.9264	-0.5364	0.0646
$\mathbf{C}$ to $\mathbf{B}$	$a_2 = 0.9385$	0.7697	-0.6906	0.1870
$\mathbf{C}$ to $\mathbf{D}$	$a_1 = 0.9385$	0.7697	-0.6906	0.1870
$\mathbf{D}$ to $\mathbf{A}$	$a_2 = 0.6800$	2.0415	-0.5539	0.0990
$\mathbf{D}$ to $\mathbf{C}$	$b_1 = 4.1557$	5.9264	-0.5364	0.0646

Supplementary Table 3: **Controlling a three-node GRN system.** For the three-node GRN system in the main text, the underlying attractor network has eight nodes, denoted as $\mathbf{A}$, $\mathbf{B}$, $\ldots$, and $\mathbf{H}$, respectively. The first column specifies all possible elementary controls among the nodes in the attractor network. The second column shows the specific coupling parameter that needs to be adjusted to realize the corresponding elementary control and the critical coupling strength. The remaining columns show the power-law scaling parameters α and β, as well as the fitting error MSE of the minimum control time τ_{m} associated with each elementary control.

Path	Critical coupling strength	α	β	MSE
$\mathbf{A}$ to $\mathbf{D}$	$a_1 = 6.0540$	10.6386	-0.5326	0.5491
$\mathbf{A}$ to $\mathbf{D}$	$b_1 = 2.7534$	6.1863	-0.5682	0.2130
$\mathbf{A}$ to $\mathbf{D}$	$c_1 = 0.1872$	0.4634	-0.7315	0.0100
$\mathbf{A}$ to $\mathbf{E}$	$a_3 = 0.1872$	0.4634	-0.7315	0.0100
$\mathbf{A}$ to $\mathbf{E}$	$b_3 = 2.7534$	6.1863	-0.5682	0.2130
$\mathbf{A}$ to $\mathbf{E}$	$c_3 = 6.0540$	10.6386	-0.5326	0.5491
$\mathbf{B}$ to $\mathbf{A}$	$a_2 = 0.1011$	0.0487	-0.9492	0.0090
$\mathbf{B}$ to $\mathbf{A}$	$b_2 = 1.0183$	1.0208	-0.8049	0.2461
$\mathbf{B}$ to $\mathbf{A}$	$c_2 = 0.1011$	0.0487	-0.9492	0.0090
$\mathbf{B}$ to $\mathbf{C}$	$a_1 = 1.0183$	1.0208	-0.8049	0.2461
$\mathbf{B}$ to $\mathbf{C}$	$b_1 = 0.1011$	0.0487	-0.9492	0.0090
$\mathbf{B}$ to $\mathbf{C}$	$c_1 = 0.1011$	0.0487	-0.9492	0.0090
$\mathbf{B}$ to $\mathbf{F}$	$a_3 = 0.1011$	0.0487	-0.9492	0.0090
$\mathbf{B}$ to $\mathbf{F}$	$b_3 = 0.1011$	0.0487	-0.9492	0.0090
$\mathbf{B}$ to $\mathbf{F}$	$c_3 = 1.0183$	1.0208	-0.8049	0.2461
$\mathbf{C}$ to $\mathbf{D}$	$a_2 = 2.7534$	6.1863	-0.5682	0.2130
$\mathbf{C}$ to $\mathbf{D}$	$b_2 = 6.0540$	10.6386	-0.5326	0.5491
$\mathbf{C}$ to $\mathbf{D}$	$c_2 = 0.1872$	0.4634	-0.7315	0.0100
$\mathbf{C}$ to $\mathbf{G}$	$a_3 = 2.7534$	6.1863	-0.5682	0.2130
$\mathbf{C}$ to $\mathbf{G}$	$b_3 = 0.1872$	0.4634	-0.7315	0.0100
$\mathbf{C}$ to $\mathbf{G}$	$c_3 = 6.0540$	10.6386	-0.5326	0.5491
$\mathbf{D}$ to $\mathbf{H}$	$a_3 = 3.8127$	6.4535	-0.5125	0.0142
$\mathbf{D}$ to $\mathbf{H}$	$b_3 = 3.8127$	6.4535	-0.5125	0.0142

**E** to **H**	$b_1 = 3.8127$	6.4535	-0.5125	0.0142
**E** to **H**	$c_1 = 3.8127$	6.4535	-0.5125	0.0142
**F** to **E**	$a_2 = 0.1872$	0.4634	-0.7315	0.0100
**F** to **E**	$b_2 = 6.0540$	10.6386	-0.5326	0.5491
**F** to **E**	$c_2 = 2.7534$	6.1863	-0.56823	0.2130
**F** to **G**	$a_1 = 6.0540$	10.6386	-0.5326	0.5491
**F** to **G**	$b_1 = 0.1872$	0.4634	-0.7315	0.0100
**F** to **G**	$c_1 = 2.7534$	6.1863	-0.5682	0.2130
**G** to **H**	$a_2 = 3.8127$	6.4535	-0.5125	0.0142
**G** to **H**	$b_2 = 3.8127$	6.4535	-0.5125	0.0142

Supplementary Table 4: **Computational setting and cost for constructing the attractor networks.** The first row specifies the systems. The second row shows the number of attractors in the system under the original parameter setting. The third row illustrates the method of calculation. The fourth row shows the number of coupling parameters tested. The fifth row shows the number of parameters that can be exploited to achieve control. The sixth row shows the computational time for each method. The software and computers for the T-cell simulation are MATLAB 2012a, 3.4GHz, Intel Core i7, Win7, while those for the two- and three-node GRNs calculations are: MATLAB 2015b, 2.9 GHz, Intel Core i7, OS X.

System	T-cell	Two-node GRN	Three-node GRN
Attractors	3	4	8
Calculation Methods	Bisection Search	Continuation	Continuation
Coupling Parameters	195	4	9
Control Parameters	48	4	9
Computation Time	4.61 hours	64.60 seconds	21.87 minutes

Supplementary Notes

Supplementary Note 1: Control of T-cell system. The success of our control scheme relies on one important condition: the underlying dynamical system is capable of responding to control perturbation in the sense that parameter perturbation can lead to dramatic changes in the system's attractors. A "rule of thumb" to determine if a dynamical system satisfies this condition can be, as follows. Given a computational model of the GRN to be controlled, we can increase the perturbation to an accessible control parameter (either activation or inhibition) to test if the corresponding attractor disappears. If it turns out impossible for the system to escape from the attractor with single parameter perturbation, multiple parameter perturbation should be tested - see Supplementary Note 4.

In the main text, we address the issue of control constraint by focusing on the minimum control time τ associated with two edges in the T-cell system as examples. Here, in Supplementary Table 1 we list the results from tuning all the identified edges that can steer the system from a cancerous state to the normal state, which include the critical coupling strength $\mu_{\rm c}$ for each edge, the power-law scaling parameters α and β for the minimum control time [$\tau_{\rm m} = \alpha \cdot |\mu_{\rm n} - \mu_{\rm c}|^{\beta}$, Eq. (2) in the main text]. The MSEs in the fitting are also listed. Additionally, in the Boolean model, the logic relationship for all the multiple in-edges towards one given node is identically "AND". Thus, when translating the Boolean model to the continuous-time model, all the in-edges share the same parameter values of $\mu_{\rm c}$, α, and β.

We also test the case of driving the system from a normal state to a cancerous state. In particular, each of the two experimentally adjustable parameters, the edge from node "Caspase" to "Apoptosis" and the self edge of "Apoptosis," can be perturbed for the control. We find that, for each control parameter, the relationship between its strength and the minimal control time also follows an algebraic scaling law with the scaling parameters: $\mu_{\rm c} \approx 0.8778$, $\alpha \approx 1.1626$, and $\beta \approx -0.6268$ [equation (2) in the main text]. The control result is in accordance with the clinical studies revealing that the T-LGL leukemia disease results from dysregulation of apoptosis [1,2].

Supplementary Note 2: Algorithms to find attractors. Given a nonlinear dynamical network, the following procedure can be used to locate all the attractors.

1. For a given parameter set, we define the search space according to the maximum and minimum possible values of each state variable. For a GRN, the maximal value of each steady state is the activation rate divided by the degradation rate whereas its minimum value is zero. For example, for our two-node GRN model, we obtain that the maximal initial value for x_1 is $(a_1 + b_1) \cdot k^{-1}$ under the assumption that the leakage term is negligible.

2. Divide the phase space into a grid to generate a large number of initial conditions (grid points). Evolve each initial condition under the system dynamics to determine the final attractors of the system. Increase the grid resolution until no new attractors appear. For example, for our two-node GRN, the initial conditions are chosen from a 11×11 grid in the two-dimensional phase space region determined by the respective ranges of the dynamical variables. There are then 121 different initial conditions, which lead to four distinct attractors. Doubling the grid resolution results in no new attractors, enabling us to conclude that there are four distinct attractors.
3. For each attractor, calculate the eigenvalues of Jacobian matrix to determine its relative stability.

Supplementary Note 3: Parameter control method in stochastic model. Our nonlinear control framework is also applicable to stochastic systems. To demonstrate this, we convert the ODE model of two-node system [Eq. (3) in the main text] to a set of Langevin equations for biochemical reactions and use the Gillespie algorithm to approximate the stochasticity [1]. Supplementary Fig. 1 shows a particular control process from attractor $\mathbf{A}$ to $\mathbf{B}$. As discussed in the main text, $\mathbf{A}$ has low abundance in both x_1 and x_2, and $\mathbf{B}$ has high abundance in x_1 and low abundance in x_2. From the attractor network in Fig. 5(d) of the main text, we see that by increasing a_1, we can drive the system from $\mathbf{A}$ to $\mathbf{B}$. Thus, we first set the system in $\mathbf{A}$ with $a_1 = 1.0$. At $t = 60$, we increase a_1 to 1.5, and at $t = 120$, we change a_1 back to 1.0. We see that, for $t > 120$ when the perturbation on a_1 has been withdrawn, the system spontaneously evolves into the attractor $\mathbf{B}$.

Supplementary Note 4: Control based on multiple parameters. To figure out the optimal parameter combinations for controlling nonlinear networks of even moderate size is computationally prohibitive at the present. However, for small networks, this can be done. For example, for the three-node GRN system studied in this paper, multiple parameter control is needed to induce certain state transition, e.g., from attractor $\mathbf{H}$ to attractor $\mathbf{B}$ in Fig. 7 in the main text. However, for the T-cell network, there are several dozens tunable edges. A surprising finding is that, because of the simplicity of the attractor network, it is only necessary to apply perturbation to any one of the 48 tunable edges to realize control.

It is interesting to note that, the sequential combination of control dosage guided by the attractor network may be regarded as a kind of control based on parameter combination. For example, it has been known that the human embryonic stem cells (hESCs) can be differentiated to a pancreatic fate under stepwise exposures to different signaling factors [2, 3]. However, the functional pancreatic fate would not occur if the differentiation steps are permuted. The non-interchangeable sequence can be understood using the concept of the attractor network, where one parameter modification represents one stage protocol. For example, in Fig. 5(d) in the main text, the hESCs correspond to attractor $\mathbf{A}$. By increasing the value of a_1 (stage 1) and then that of b_2 (stage 2), we can drive the system to attractor $\mathbf{C}$. However, if we increase b_2 first (stage 2 first), we will not be able to drive the system to attractor $\mathbf{C}$ by increasing the value a_1 (stage 1) [see also the top branch of attractor $\mathbf{D}$ in Fig. 5(a) in the main text]. We emphasize that these results do not imply that the

sequential combination method is restricted to perturbation to one parameter at a time. Indeed, recent works suggested that simultaneous therapy with two drugs can be much more effective than sequential therapy [4].

Supplementary Note 5: Algorithmic complexity and computational cost. In the attractor network, a link is determined when the undesired attractor vanishes upon application of control parameter perturbation of certain magnitude (within some predefined range). For each attractor, we perform a bisecting search for all the tunable parameters to establish the possible links in the attractor network. In each search, the number of checks to see if the attractor has disappeared is $\log_2(1/\Delta)$, where Δ is the accuracy in the estimate of the critical perturbation amplitude. The total number of bisecting searches is the number of attractors, N_{S}, multiplying the number of tunable parameters, N_{L}. In a nonlinear system, the number of attractors depends on the system size. For example, for a boolean network, there are 2^N possible states. While the actual number of attractors can be much less than 2^N, it depends on the network size N. The number of control parameters is the number of controllable links. Assuming that the network is sparse (as in many biological networks), the total number of checks is $\approx \log_2(1/\Delta) \times N_S \times \rho N^2$, where ρ is the connection probability. The bisecting searching needs to be performed once for a sufficiently large time duration τ of the control. The reason is that, if the control is achievable for a link for a longer duration, the same control can be realized for a shorter duration but with a larger control amplitude.

For nonlinear and complex dynamical systems, the relationship between control perturbation and escaping from an attractor can be discontinuous and/or non-monotonic. In such a case, a "blind" application of the bisecting search to build up the attractor network and to estimate the computational cost may not be effective or even fail. This difficulty, however, can be overcome by using the method of parameter continuation with multiple initial conditions, which is standard in generating and analyzing the bifurcation diagrams of nonlinear dynamical systems. Especially, one can choose a small number of random initial conditions and a small set of parameters in a physically/biologically meaningful range, and determine the distinct attractors that the system possesses. The result will be a global picture of the possible attractors of the system in a small number of parameter intervals, which will facilitate greatly the computational task.

Supplementary Note 6: Phase diagram. A phase diagram illustrates how different choices of the parameters affect the system's stable asymptotic states (attractors). Supplementary Fig. 2 shows the phase diagram of the two-node GRN system with respect to variations in a_1 and b_2. Each point in the diagram represents, for the particular combination of values of a_1 and b_2, the whole set of possible attractors of the system. There are seven possible combinations of attractors in the diagram. As shown in Fig. 5(d) in the main text, if we set out to control the system from attractor $\mathbf{A}$ to attractor $\mathbf{C}$, we can first steer the system from $\mathbf{A}$ to $\mathbf{B}$ through perturbation on a_1 and then drive the system from $\mathbf{B}$ to $\mathbf{C}$ by tuning b_2. Note that the sequence of a_1 and b_2 is important here: if we first perturb b_2, the system in attractor $\mathbf{A}$ will be driven to attractor $\mathbf{D}$. When this happens, the system will remain in $\mathbf{D}$, regardless of any additional parameter adjustments, rendering unrealizable the control goal to drive the system into attractor $\mathbf{C}$. This scenario can also be seen from the phase diagram in Supplementary Fig. 2. In particular, starting from the dark red region, with the

particular combination of parameters, there are 4 attractors: $\mathbf{A}$, $\mathbf{B}$, $\mathbf{C}$ and $\mathbf{D}$. As we increase a_1 and set the parameters to the red area, attractor $\mathbf{A}$ disappears and its basin is merged into that of one of the remaining three attractors ($\mathbf{B}$, $\mathbf{C}$, and $\mathbf{D}$). From Fig. 4(e) in the main text, we see that $\mathbf{A}$ migrates into $\mathbf{B}$. Thus, when we withdraw the perturbation on a_1 and turn on the perturbation on b_2, the possible attractors of system are $\mathbf{C}$ and $\mathbf{D}$ (the light green area). Figure 5(d) in the main text also indicates that $\mathbf{B}$ migrates to $\mathbf{C}$. We note that, in the phase diagram, it is difficult to distinguish into which state $\mathbf{A}$ merges, but this can be readily accomplished through the attractor network in Fig. 5(d) in the main text, which indicates unequivocally that the basin of $\mathbf{A}$ is absorbed into that of $\mathbf{D}$.

Supplementary Note 7: Termination criteria and calculation of control strength and minimum control time. For all systems studied in this paper, of particular importance to control is the relationship between control strength and minimum control time. The attractor network needs to be constructed first based on the procedures described in **Methods** in the main text. From the attractor network, we can obtain the control parameter for the transition between any two states. For each control parameter, we choose several different values which can realize the control and use a *bisecting search* to find the minimum control time. We then apply linear regression to the data on a double logarithmic scale to find the value of μ_{c}. In the bisecting search process, the time interval is chosen to be $t_{\mathrm{s}} = 10^{-2}$. For the T-cell and two-node GRN systems detailed in the main text, we choose T_{f} to be 100. For the three-node GRN system, we choose T_{f} to be 1000. The results for each system are illustrated in Supplementary Table 1-3 and the following notes.

Supplementary Note 8: Parameter control in the two-node and three-node GRNs. For the two-node GRN system [Eq. (3) in the main text], the corresponding attractor network consists of four attractors, denoted by $\mathbf{A}$, $\mathbf{B}$, $\mathbf{C}$, and $\mathbf{D}$, respectively. All parameter controls to realize the transitions among them are given in Supplementary Table 2, together with the critical coupling strength, the minimum control time, and so on.

Similarly, for the three-node GRN system [Eq. (4) in the main text], eight attractors($\mathbf{A}$, $\mathbf{B}$, $\mathbf{C}$, $\mathbf{D}$, $\mathbf{E}$, $\mathbf{F}$, $\mathbf{G}$ and $\mathbf{H}$,) constitute the attractor network. Supplementary Table 3 illustrates the critical coupling strength and the minimal control time to realize control.

Supplementary References

1. Gillespie, D. T. Exact stochastic simulation of coupled chemical reactions. *J. Phys. Chem.* **81**, 2340–2361 (1970).

2. D'Amour, K. A. *et al.* Production of pancreatic hormone–expressing endocrine cells from human embryonic stem cells. *Nat. Biotechnol* **24**, 1392–1401 (2006).

3. Kroon, E. *et al.* Pancreatic endoderm derived from human embryonic stem cells generates glucose-responsive insulin-secreting cells in vivo. *Nat. Biotechnol* **26**, 443–452 (2008).

4. Bozic, I. *et al.* Evolutionary dynamics of cancer in response to targeted combination therapy. *eLife* **2**, e00747 (2013).
